# An orthogeriatric service can reduce prolonged hospital length of stay in hospital for older adults admitted with hip fractures: a monocentric study

**DOI:** 10.1007/s40520-023-02616-3

**Published:** 2023-11-14

**Authors:** Radcliffe Lisk, Keefai Yeong, David Fluck, Jonathan Robin, Christopher Henry Fry, Thang Sieu Han

**Affiliations:** 1 Department of Orthogeriatrics, Ashford and St Peter’s NHS Foundation Trust, Guildford Road, Chertsey, KT16 0PZ Surrey UK; 2 Department of Cardiology, Ashford and St Peter’s NHS Foundation Trust, Guildford Road, Chertsey, KT16 0PZ Surrey UK; 3 Department of Acute Medicine, Ashford and St Peter’s NHS Foundation Trust, Guildford Road, Chertsey, KT16 0PZ Surrey UK; 4 School of Physiology, Pharmacology and Neuroscience, University of Bristol, Bristol, BS8 1TD UK; 5 Department of Endocrinology, Ashford and St Peter’s NHS Foundation Trust, Guildford Road, Chertsey, KT16 0PZ Surrey UK; 6 Institute of Cardiovascular Research, Royal Holloway, University of London, Egham, TW20 0EX Surrey UK

**Keywords:** Hip fractures, Time to discharge, Discharge destinations, Health economics

## Abstract

**Background:**

The Blue Book (2005), recommended guidelines for patients care with fragility fractures. Together with introduction of a National Hip Fracture Database Audit and Best Practice Tariff model to financially incentivise hospitals by payment of a supplement for patients whose care satisfied six clinical standards), have improved hip fracture after-care. However, there is a lack of data-driven evidence to support its effectiveness. We aimed to verify the impact of an orthogeriatric service on hospital length of stay (LOS)—duration from admission to discharge.

**Methods:**

We conducted a repeated cross-sectional study over a 10 year period of older individuals aged ≥ 60 years admitted with hip fractures to a hospital.

**Results:**

Altogether 2798 patients, 741 men and 2057 women (respective mean ages; 80.5 ± 10.6 and 83.2 ± 8.9 years) were admitted from their own homes with a hip fracture and survived to discharge. Compared to 2009–2014, LOS during 2015–2019, when the orthogeriatric service was fully implemented, was shorter for all discharge destinations: 10.4 *vs* 17.5 days (*P* < 0.001). Each discharge destination showed reductions: back to own homes, 9.7 *vs* 17.7 days (*P* < 0.001); to rehabilitation units: 10.8 *vs* 13.1 days (*P* < 0.001); to residential care: 15.4 *vs* 26.2 days (*P* = 0.001); or nursing care, 24.4 *vs* 53.1 days (*P* < 0.001). During 2009–2014, the risk of staying > 3 weeks in hospital was greater by six-fold and pressure ulcers by three-fold. The number of bed days for every thousand patients per year was also shortened during 2015–2019 by: 1665 days for discharge back to own homes; 469 days with transfer to rehabilitation units; 1258 days for discharge to residential care, and 5465 days to nursing care. Estimated annual savings (2017 costs) per thousand patients after complete establishment of the service was about £2.7 m.

**Conclusions:**

Implementation of an orthogeriatric service generated significant reductions in hospital LOS for all patients, with associated cost-savings, especially for those discharged to nursing care.

**Supplementary Information:**

The online version contains supplementary material available at 10.1007/s40520-023-02616-3.

## Introduction

Hip fractures are amongst the commonest hospital admissions and occupy more days in hospital than most other acute conditions [1]. Although there is a need for a period of recovery after a hip operation, patients with hip fractures may stay in hospital beyond their expected date of discharge. In many cases, patients are “medically-fit-for-discharge” but encounter non-clinical factors that lead to a delay. Consequently, this increases the risk of developing nosocomial complications [2–4], but also incurs excess costs to the NHS and reduces hospital capacity [5]. To address these challenges, nationwide efforts were initiated to improve management of hip fractures. In 2005, the Blue Book was published, jointly sponsored by the British Geriatrics Society and the British Orthopaedic Association, which recommended guidelines for the care of patients with fragility fractures [6]. In 2007, a national audit programme for England and Wales was launched [7], with a web-based audit tool (the National Hip Fracture Database, NHFD), created to allow hospitals to monitor the care-quality provided to individual patients and their outcomes [8]. These initiatives were followed by the introduction of the pay-for-performance model (the Best Practice Tariff for hip fractures) in 2010, to financially incentivise hospitals by payment of a supplement for each patient whose care satisfied six clinical standards (see Methods) [9]. These factors have prompted us to initiate an orthogeriatric service to comply with their recommendations to improve patient care. Since then, the influence of orthogeriatric care on hip fracture outcomes has been documented by several small studies [10–13] with short periods of observation [10, 11, 14]. Only a few studies have used data that were collected for NHFD [11]. Our recent study [15] of a greater number of patients observed over a decade showed that prolonged hospital length of stay (LOS) was reduced progressively after introduction of a fully implemented orthogeriatric service as described above. However, there are no published data on how this service influenced hospital LOS (defined as the duration of stay in hospital from the point of admission to discharge) to in patients who are discharged to different destinations. This information is important since each discharge destination faces different requirements that could determine the time to discharge. These include arrangements for carers or house modifications for those returning home. Furthermore, individuals who need greater levels of care may have to wait for the availability of a new placement such as rehabilitation unit, residential or nursing care. This study of patients admitted from home to hospital with a hip fracture, was over a 10 year period, punctuated mid-way by implementation of an orthogeriatric service. We aimed to verify the impact of an orthogeriatric service on hospital length of stay (LOS). We examined the impact of this initiative on LOS for patients who were discharged either back to their homes, or upon transfer to rehabilitation units, residential care or nursing care.

## Methods

### Milestones in a service development for hip-fracture patients

Before 2010, hip-fracture patients were cared solely by an orthopaedic team, with ad hoc review from a general medical team when required. In September 2010, Ashford and St Peter’s NHS Foundation Trust, Surrey, UK, initiated an orthogeriatric service for joint care of hip fracture patients, led by orthogeriatricians. The service was reconstructed based on the “Lean Principles” known as EQuIP (efficiency, quality, innovation, and productivity programme). Six key areas for service improvement of patients admitted with a hip fracture were set by the Best Practice Tariff criteria to achieve: i) time to surgery within 36 h from arrival in an emergency department, or time of diagnosis if an inpatient, to the start of anaesthesia; ii) admission of patients under the joint care of a consultant geriatrician and a consultant orthopaedic surgeon; iii) admission using an assessment protocol agreed by geriatric medicine, orthopaedic surgery and anaesthetic benchmarks; iv) assessment by a geriatrician in the preoperative period: within 72 h of admission; v) assessment by a postoperative geriatrician-directed multi-professional rehabilitation team; and vi) performance of fracture prevention assessments (falls and bone health) [9]. By achieving these standards of care, and setting up facilitated care pathways for these patients, especially prioritisation to theatre and integration of orthogeriatricians into the routine care for these patients, there were improvements in patient outcome and experience and reduced hospital LOS. At this time, hip-fracture patients were cared for in a geriatric ward with the orthopaedic surgeon acting as a consultative specialist, whilst the orthogeriatrician was responsible for the care of the patients, conducting two consultant ward rounds a week [16]. Since the orthogeriatric service was continually being assessed for improvement, additional components were introduced in a stepwise manner, *i.e.* not all components were implemented at once. The next major change was introduction of an orthogeriatric supportive discharge (OSD) team in 2013, conferring targeted intervention to reduce hospital LOS. In addition to the implementation of the orthogeriatric service, daily ward rounds, led by a consultant-of-the-week (the COW model for orthogeriatrics and orthopaedics), was further provided in 2016 to support continuity of care. The COW model is an integrated shared-care model [13] involving a 7 days-a-week ward round led by an orthopaedic surgeon, and an orthogeriatrician on weekdays, who work with a multi-disciplinary team of physiotherapists, nurses, occupational therapists and social service professionals [17].

### Study design, participants and setting

We conducted a study based on repeated cross-sectional design of older individuals aged ≥ 60 years [9] admitted with hip fractures to a single National Health Service hospital, *i.e.* we did not use the entire national data from NHFD. To avoid bias from patients with poorer health, such as those admitted from residence or nursing care, only patients who were admitted from their own homes and survived to discharge were analysed (5.1% of patients who died in hospital were excluded).

### Measurements

Through our participation in the NHFD Audit Programme [8, 17, 18], data from time of admission to discharge were prospectively collected by a Trauma Coordinator for all patients admitted with a hip fracture to a single hospital (Ashford and St Peter’s NHS Foundation Trust). These data comprised detailed clinical measures, including: age; sex; physical status based on the American Society of Anesthesiologists (ASA) classification; elapsed time to surgery; anaesthesia types; surgical techniques; pressure ulcers; hospital LOS, as well as discharge destination (back to patients’ own homes; rehabilitation units; residential care; or nursing care). Data were collected from 2009 to 2019 and routinely updated and checked by the orthogeriatrician to ensure completeness and accuracy.

### Categorisation of variables

The period of study was categorised into every financial year (from beginning of April 1 year to end of March next year) for initial assessment. This was followed by categorisation of the data into two separate groups (2009–2014 and 2015–2019) to reflect during and after implementation of most of the components of the orthogeriatric care service (except the COW model which was introduced in 2016). Change in discharge destination was considered for those who came from their own home before admission but transferred to places where increased care was provided, including rehabilitation units, residential home or nursing care. Pre-fracture mobility status was grouped into five categories: i) freely mobile without aids, ii) mobile outdoors with one aid, ii) mobile outdoors with two aids or frame, iv) some indoor mobility but never goes outside without help, and v) no functional mobility (using lower limbs) [8]. The last two categories were considered as a “limited pre-fracture mobility” group. Categorisation of ASA was examined in patients with grade ≥ 3 (severe systemic disease or severe systemic disease that is a constant threat to life). Delay in elapsed time to surgery was considered if hip surgery was beyond 36 h from time of admission as defined by the Best Practice Tariff criteria [8]. Prolonged LOS was defined as those staying more than 3 weeks in hospital.

### Statistical analysis

Characteristics of patients were assessed by descriptive statistics and their differences between groups were assessed by chi-squared test. Normality of LOS in hospital was examined by histograms and Kolmogorov–Smirnov (KS) test. The distribution of LOS displayed a right skewness (KS test: *P* < 0.001) (Supplementary Fig. 1). Thus differences in LOS were compared by non-parametric tests including Kruskal–Wallis test with pairwise comparisons for more than two groups, and Mann–Whitney U test or KS for two groups. The nonparametric test KS was selected in this study as it is suitable for continuous data drawn from populations with same cumulative distribution function [19]. This statistic quantifies the greatest distance between cumulative distribution function of two samples, thus allowing us to assess for differences in LOS between two study periods. Multivariable logistic regression was used to compare the risk of prolonged LOS between periods of study, presented as odds ratios (OR) and 95% confidence intervals (CI) obtained from two models: i) unadjusted, and ii) adjusted for age, sex, pre-fracture mobility and types of hip surgery (arthroplasty, intramedullary nails, sliding hip screws, total hip replacement hybrid and others). The LOS for patients being discharged to different destinations was standardised as the number of bed days per thousand patients per year. All statistical analyses were conducted using SPSS statistical software package (IBM Corp. Released 2021. IBM SPSS Statistics for Windows, Version 28.0. Armonk, NY: IBM Corp).

## Results

### General description over the period of the survey

A total of 2798 patients were admitted from their own homes with a hip fracture and survived to discharge. There were 20.3% patients older than 90 years, 25.4% with limited pre-fracture mobility, with 67.5% treated with general anaesthetics. 19.7% had elapsed time beyond 36 h, and most of the patients had arthroplasty (51.4%), followed by similar proportions receiving intramedullary nails (22.4%) and a sliding hip screw (24.7%), with only 1.6% receiving total hip replacement hybrid or other techniques. Hospital acquired pressure ulcers occurred in 3.1% of patients. The majority of patients returned to their own homes (65.1%), followed by transfer to rehabilitation units (27.6%), nursing care (4.9%) and residential care (2.4%) (Table 1).

**Table 1:** Clinical characteristics of patients during 2009–2014 and during 2015–2019

	Distribution						
	All (*n* = 2,798)		2009–2014 (*n* = 1,375)		2015–2019 (*n* = 1,423)		Chi-square test for group differences*
	n	%	n	%	n	%	*P*
Men	741	24.5	347	25.2	394	27.7	0.077
Age ≥ 90 years at operation	567	20.3	257	18.7	310	21.8	0.023
Limited pre-fracture mobility	710	25.4	264	19.3	446	31.3	< 0.001
ASA classification grade ≥ 3	1156	45.1	381	33.1	775	55.8	< 0.001
*Anaesthesia types*							
Spinal block with general anaesthetics	1095	42.4	371	31.8	724	51.2	< 0.001
Spinal block only	495	19.2	283	24.3	212	15.0	< 0.001
General anaesthetics only	648	25.1	178	15.3	470	33.2	< 0.001
Others	344	13.3	335	28.7	9	0.6	< 0.001
*Elapsed time to surgery*							
≥ 36 h	552	19.7	334	24.3	218	15.3	< 0.001
*Surgical techniques*							
Arthroplasty	1436	51.4	717	52.2	719	50.5	0.195
Intramedullary nail	626	22.4	207	15.1	419	29.4	< 0.001
Sliding hip screw	690	24.7	448	32.6	242	17.0	< 0.001
Total hip replacement hybrid and others	44	1.6	1	0.1	43	3.0	< 0.001
Hospital acquired pressure ulcers	87	3.1	62	4.5	25	1.8	< 0.001
*Discharge destinations*							
Home	1822	65.1	969	70.5	853	59.9	< 0.001
Rehabilitation unit	772	27.6	286	20.8	486	34.2	0.042
Residential care	66	2.4	25	1.8	41	2.9	< 0.001
Nursing care	138	4.9	95	6.9	43	3.0	< 0.001

Comparing differences in distribution between 2009–2014 and 2015–2019

*ASA*, American Society of Anesthesiologists

**Fig. 1:**
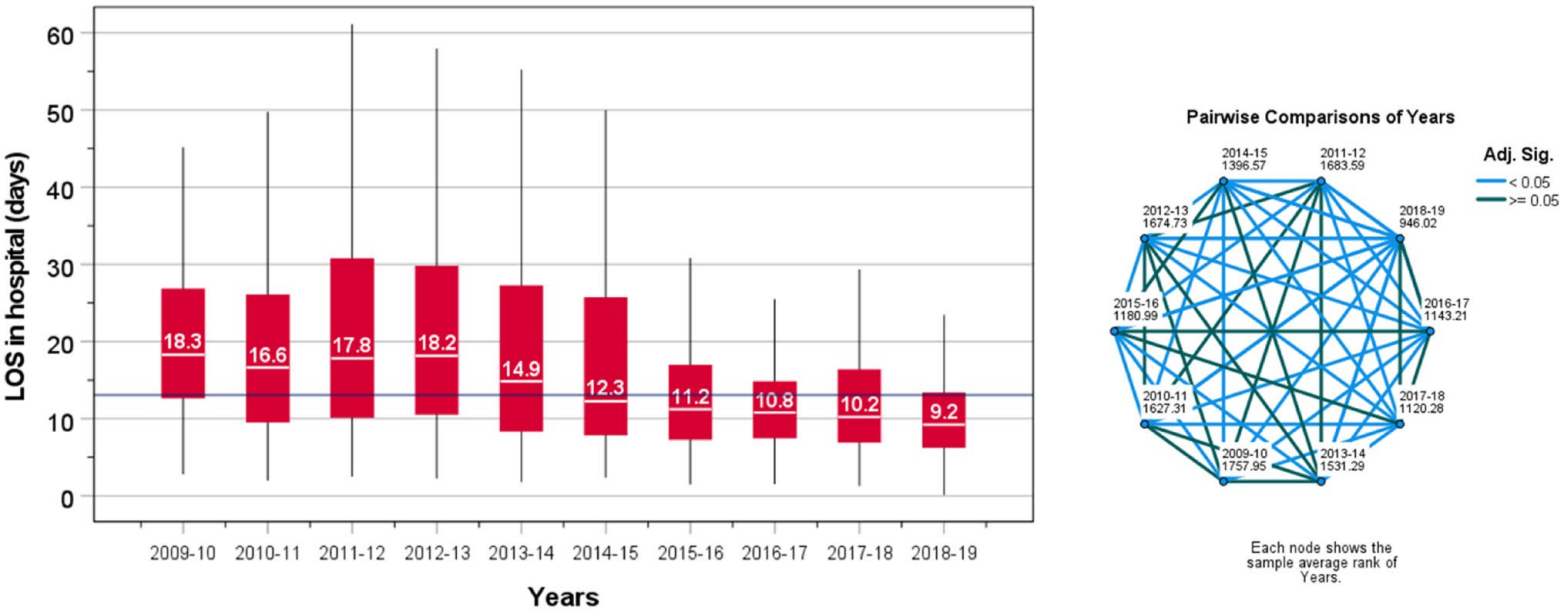
Length of stay in hospital according to every 12 month (financial year) recruitment for the entire sample and corresponding pairwise comparison test between years of recruitment using Kruskal–Wallis: light blue lines indicate significant differences between each pair (*P* < 0.05) and dark blue lines indicate no differences

**Fig. 2:**
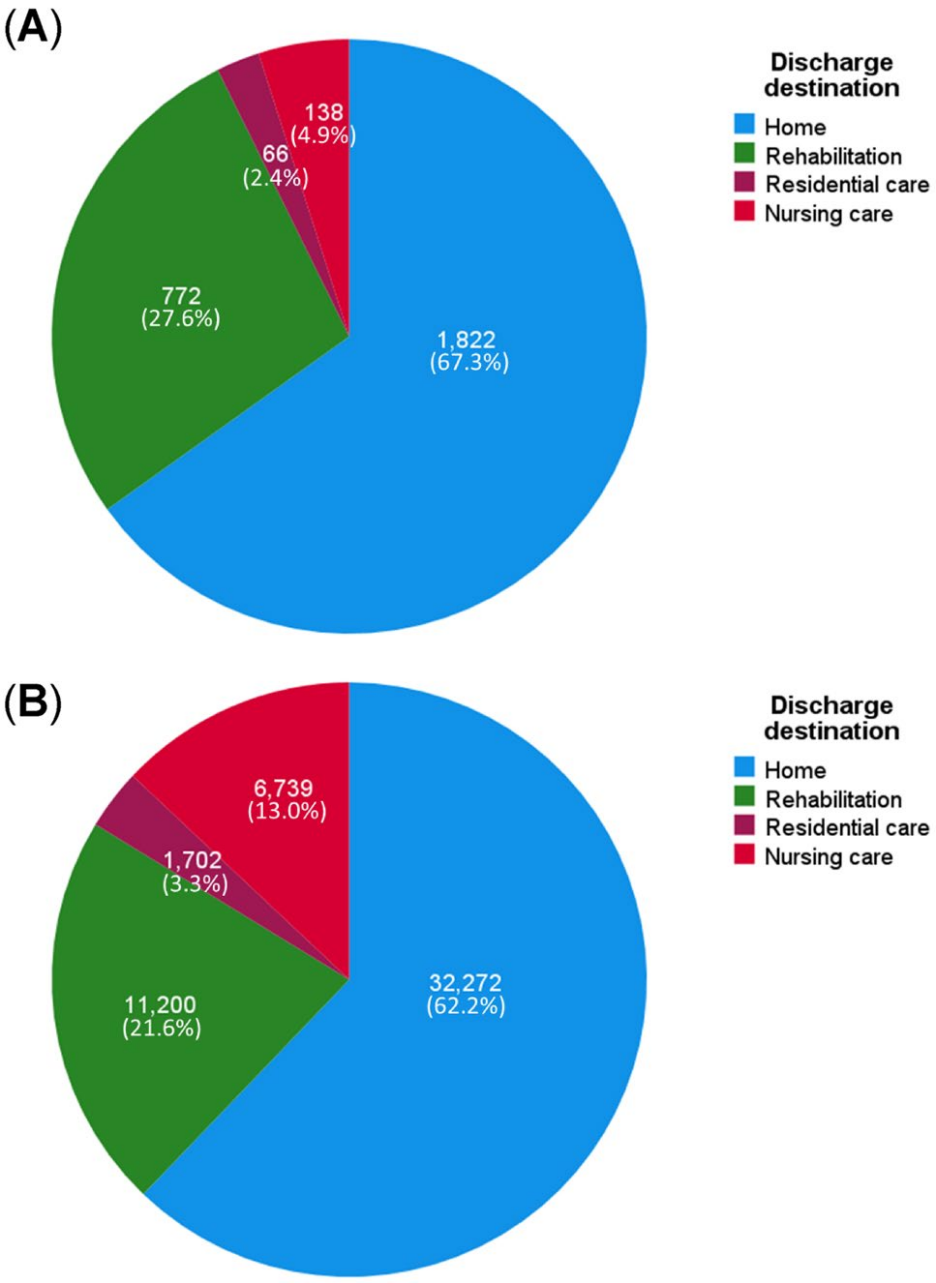
Distribution of total number of patients **A** and LOS in hospital **B** according to discharge destination (*n* = 2,798)

During the first 4 years of the study, the median LOS was relatively steady between 16.6 and 18.3 days, which dropped to 14.9 the following year. A further decrease to 12.3 days in 2014–2015 was observed, which continued to decrease year on year to the end of the study. By the last year of study, the median LOS was 9.2 days, compared to 18.3 days at the beginning of the study (Fig. 1). There were 67.3% returned to their own homes and 27.6% were newly transferred to rehabilitation units, 2.4% to residential care, and 4.9% to nursing care (Fig. 2A). The combined LOS in hospital for all patients was 51,913 days, of which 32,272 days (62.2%) were spent by patients who eventually returned to their own homes; 11,200 days (21.6%) by patients newly transferred to rehabilitation units; 1702 days (3.3%) moved to residential care; and 6739 days (13.0%) for those admitted to nursing care (Fig. 2B), The median (IQR) LOS in hospital for those discharged back to home was 13.1 days (7.9–23.0), for those discharged to rehabilitation units was 11.8 days (8.2–17.5), to residential care was 21.6 days (11.3–34.0), and to nursing care was 38.6 days (23.3–67.1) (Supplementary Fig. 2). Thus, the smallest proportion of patients, who were eventually discharged to nursing care, were those who spent the longest period in hospital.

### Impact of orthogeriatric care service

**Fig. 3:**
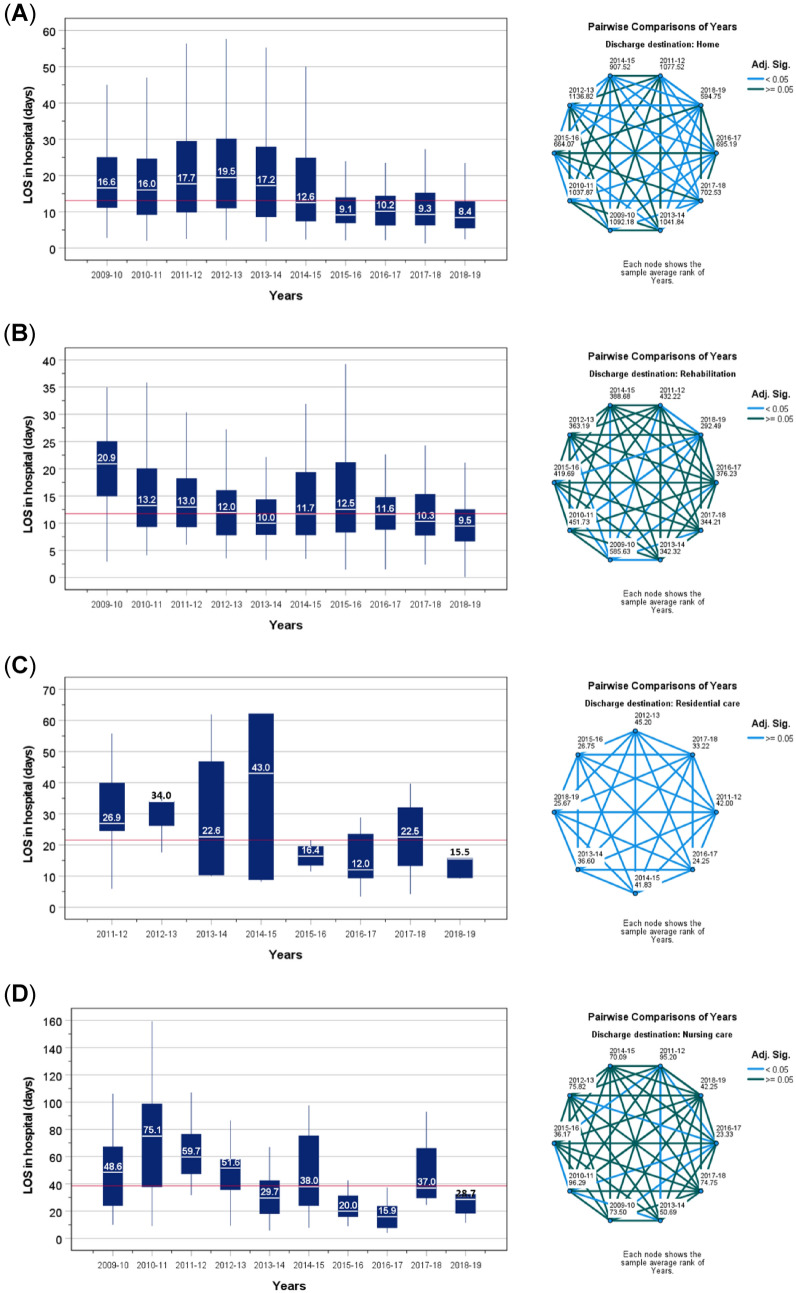
Length of stay in hospital according to every 12 month (financial year) recruitment amongst patients who were discharged back to own home (**A**), or to new a destination: rehabilitation units (**B**), residential care (**C**), or nursing care (**D**). Pairwise comparisons years of recruitment was conducted by Kruskal–Wallis test: light blue lines indicate significant differences between each pair (*P* < 0.05) and dark blue lines indicate no differences

Detailed examination on individual discharge destinations showed the reduction of LOS for all discharge destinations was most marked after most components of the orthogeriatric service were implemented by 2015, with the additional COW model introduced in 2016. Compared to patients admitted during 2009–2014 (*n* = 1375), those admitted during 2015–2019 (*n* = 1423) were: older by 1 year (95%CI = 0.3–1.7, *P* = 0.002): mean ± SD 83.0 ± 8.6 years vs 81.9 ± 10.2 years); had higher proportions of very old age (≥ 90 years); limited pre-fracture mobility; general anaesthetics for hip operation; treatment with intramedullary nails; and discharge to a rehabilitation unit or residential care. However, there were lower rates of elapsed time to surgery beyond 36 h; treatment with sliding hip screw; hospital acquired pressure ulcers; and discharge to own homes or nursing care (Table 1). During the latter half of the study (2015–2019), the LOS were mostly below the group median values for patients being discharge to any of their eventual destinations (Fig. 3). The median hospital LOS for all discharge destinations was between 14.9 and 18.3 days during 2009–2014 and fell to below the grand median value of the entire sample (13.1 days) during 2015–2019).

Examination of the distribution of patients according to the LOS in hospital for all patients showed that compared to the period of 2009–2014, there were relatively greater numbers of patients being discharged earlier during when the orthogeriatric service was fully implemented in the period 2015–2019 (Supplementary Fig. 3). Compared to LOS during 2009–2014, the LOS during 2015–2019 was shorter for all discharge destinations (Mann–Whitney U tests): 10.4 *vs* 17.5 days (*P* < 0.001). Corresponding values for sub-group destinations were: returning to own homes 9.7. *vs* 17.7 days (*P* < 0.001); transfer to rehabilitation units, 10.8 *vs* 13.1 days (*P* < 0.001); discharge to residential care; 15.4 *vs* 26.2 days (*P* = 0.001); or nursing care: 24.4 *vs* 53.1 days (*P* < 0.001).

Multivariable logistic regression analysis with adjustments for age, sex, pre-fracture mobility and types of hip surgery showed that compared with the 2015–2019 group (reference), the 2009–2014 group had greater risk of prolonged LOS in hospital (> 3 weeks): adjusted OR (95%CI) = 4.59 (3.76–5.61) amongst all discharge destinations; and for those destined back to their own homes: OR = 5.92 (4.52–7.76); to rehabilitation: OR = 1.72 (1.14–2.58), residential care: OR = 6.01 (1.58–22.96); and to nursing care: OR = 7.33 (2.78–19.28). For all patients discharged to any destination, the 2009–2014 group also had high risk of sustaining pressure ulcers: OR = 3.07 (1.87–5.04) (Table 2).

**Table 2:** Multivariable logistic regression analysis to assess the risk of prolonged LOS in hospital (> 3 weeks) and pressure ulcers during 2009–2014 period compared with 2009–2014 period (reference) amongst patients being discharged to different destinations

	Unadjusted			Adjusted for age, sex, pre-fracture mobility and types of hip surgery		
Discharge destinations	OR	95%CI	*P*	OR	95%CI	*P*
All discharge destinations	3.35	2.81–4.00	< 0.001	4.59	3.76–5.61	< 0.001
Back to own homes	3.85	3.07–4.83	< 0.001	5.92	4.52–7.76	< 0.001
Rehabilitation units	1.58	1.08–2.31	0.018	1.72	1.14–2.58	0.009
Residential care	4.028	1.37–11.77	0.011	6.01	1.58–22.96	0.009
Nursing care	6.05	2.53–14.43	< 0.001	7.33	2.78–19.28	< 0.001
*Complications*						
Pressure ulcers	2.64	1.65–4.23	< 0.001	3.07	1.87–5.04	< 0.001

OR, odds ratios; CI, confidence intervals

**Fig. 4:**
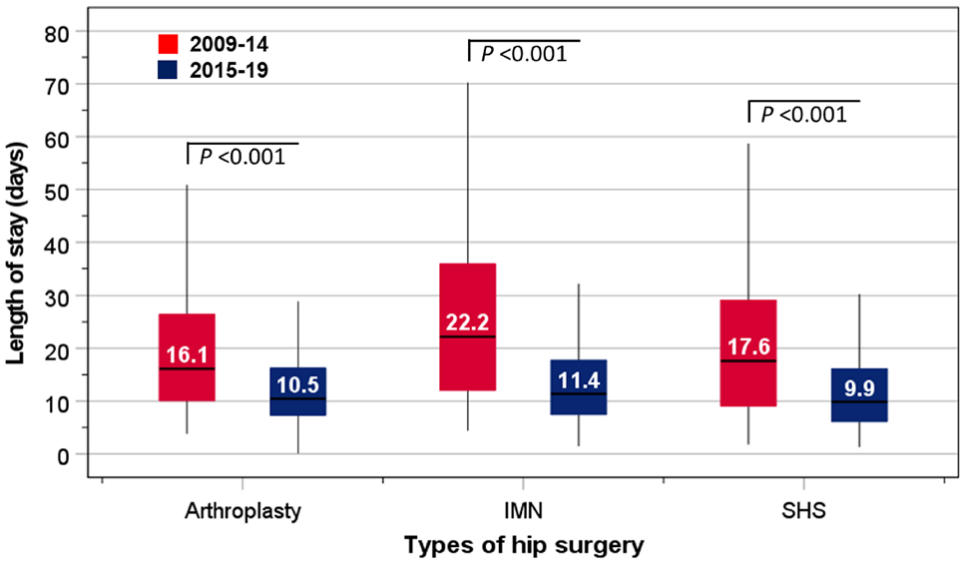
Length of stay in hospital according to different types of surgical procedures (arthroplasty; IMN, intramedullary nails; SHS, sliding hip screws) performed during 2009–14 and 2015–2019. Group differences were compared using Mann–Whitney U tests

Compared with the 2009–2014 period, LOS during the 2015–2019 period was significantly lower for those undergoing arthroplasty: 16.1 vs 10.5 days, *P* < 0.001), intramedullary nails (22.2 vs 11.4 days, *P*< 0.001), and sliding hip screws (17.6 vs 9,9 days, *P* < 0.001). There were too few patients undergoing total hip replacement for analysis (Fig. 4).

Standardised data for LOS per thousand were calculated for comparison between groups. Compared to 2009–2014, the number of bed days for every 1000 patients per year during the latter period (2015–2019) was reduced by 1665 days for those discharged back to own home; by 469 days for those transferred to rehabilitation units; 1258 days for those newly discharged to residential care and 5465 days for those requiring nursing care. The corresponding figures for types of surgery including arthroplasty, intramedullary nails, and sliding hip screws were 1496, 2480, and 1895 bed days (Table 3).

**Table 3:** Number of bed days occupied by patients in 2009–2014 and in 2009–2014. Data were standardised for length of stay per thousand patients per year for comparison

	Total length of stay (days/number of patients/5 yr)		Standardised data of length of stay (days/one thousand patients/yr)			
	2009–2014	2015–2019	2009–2014	2015–2019	Reduction	Savings*
*Discharge destinations*						
Back to own homes	20,941/969	11,332/853	4322	2657	**1665**	**£495,507**
Rehabilitation units	4576/286	6624/486	3200	2732	**469**	**£140,419**
Residential care	742/25	959/41	5.938	4680	**1258**	**£376,645**
Nursing care	5448/95	1291/43	11,469	6005	**5465**	**£1,636,221**
*Types of surgery*						
Arthroplasty	15,529/717	10,196/719	4332	2836	**1496**	**£447,902**
Intramedullary nail	5696/207	6334/419	5503	3023	**2480**	**£742,512**
Sliding hip screw	10,381/448	3314/242	4634	2739	**1895**	**£567,363**

Bold type to highlight the net reduction and savings

*Saving based on calculations of the estimated cost to the NHS of £299.4 per bed day per person

## Discussion

In this study of patients admitted with hip fractures over a 10 year period, which included initially the implementation of an orthogeriatric service, there was a profound impact on patients’ time to discharge from hospital, as indicated by the LOS. After complete implementation of this service, the number of bed days was significantly reduced in all patients including those who returned to their own home, newly transferred to rehabilitation units, but most markedly to those newly discharged to residential or nursing care. These findings support the value of an orthogeriatric service for managing patients admitted with hip fractures and could be used as a service model for other specialties.

A previous study in the US showed an average of 35% reduction in hospital LOS over a period of 11 years for patients admitted with hip fractures [20]. However, as far as we are aware, there exists no literature on LOS to various discharge destinations amongst hip fracture patients who are admitted from homes. Our observations indicate that the application of an integrated orthogeriatric service could overcome barriers to timely discharge of “medically-fit” patients, particularly those who require a new placement such as residential or nursing care. Since patients’ underlying characteristics such age, sex, frailty and health status, as well types of surgical procedures may influence LOS in hospital, multivariable logistic regression was conducted to take these factors into account. The results confirmed that during the earlier period of study (2009–2014) was associated with an increased risk of prolonged LOS (staying > 3 weeks in hospital). These findings are therefore robust. A reduction of hospital patient LOS has many benefits and timely discharge reduces the risk of death and readmission after hospital discharge [5]. Furthermore, each extra day in hospital increases the accumulative risk of losing muscle strength by about 5% [3] through nosocomial infections, pressure ulcers, malnutrition and delirium [4, 5]. Our findings of a three-fold increase in the risk of pressure ulcers during the 2009–2014 period compared to 2015–2019 period are therefore, supportive of those previous reported. These problems further accentuate the delay to discharge, leading to a vicious cycle of even greater prolonged hospitalisation, and further adding to healthcare service costs. It is also possible that nosocomial complications acquired from prolonged LOS due to non-clinical factors will increase the need for a higher level of care on discharge, resulting in a change of discharge destination that provides to a high-level of care. Thus, solving barriers to discharge and improving time to surgery will have a beneficial effect. It fits well with the notion that the only patients in hospital should have a reason to be there, as adjudged by the criteria to admit and reason to reside [21].

The estimated per-patient cost of excess bed days in 2017 was £2096/week for non-elective in-patients [22]. The small number of hip-fracture patients (4.9% of total) waiting for a place in nursing care occupied relatively more bed days (13% of all LOS) than those waiting for other discharge destinations. Year-on-year analysis in the period 2015–2019, when the orthogeriatric service was fully implemented, showed that the LOS was halved when compared to 2009–2014. We estimate that for every thousand hip-fractures requiring admission to nursing care this would reduce in-hospital costs by £1,636,221 (€1,876,509). According to these data, the total savings for all hip fractures per thousand patients per year are about £2,650,000 (€3,039,167). These observations are similar to a smaller Portuguese study of hip-fracture patients with an overall discharge delay of 22.3% adding 11.2–30.7% of total costs (€2352–9317 = £2026–8025 per patient) [23]. We have also demonstrated the amounts of money saved for each type of surgical procedure during the 2015–2019 period to be £447,902 (€513,678), £742,512 (€851,554), and £567,363 (€650,683) for arthroplasty, intramedullary nails, and sliding hip screws, respectively. We recognise that the overall cost-savings to health services are far more complex, involving many more aspects such as family members, social services, medications and hospital visits. We found that proportionally more patients were transferred to rehabilitation units before being discharged home, which would incur additional costs. However, the costs of rehabilitation are substantially cheaper than a hospital stay, so there remains an overall cost-saving [24].

Furthermore, compared to the first period of study (2009–2014), between 2015 and 2019, when the orthogeriatric service was fully implemented, more hip operations were also performed on higher risk patients, including those over 90 years with high ASA scores (see Table 1). Previous study of this cohort of patients showed that over this time, the use of arthroplasty did not change, but there was an increased use of intramedullary nails and a decrease of sliding hip screws, which may reflect changes in surgical practice or preference [15]. There was also a significant reduction in hospital acquired pressure ulcers (see Table 1), which highlighted the positive impact of reducing time to discharge. However, to maintain and improve current discharge planning, it is necessary to have adequate funding for discharge options such as nursing and community care. Recently, the government announced a £500 million fund to help with adult social care discharge, of which £200 million will be distributed to local authorities, and £300 million to integrated care boards, targeted at those areas experiencing the greatest discharge delays [25]. It appears that implementation of the orthogeriatric service had a progressive impact on the reduction of LOS. The reason for choosing the two groups at 2009–2014 and 2015–2019 in the study was based on the approximate time the orthogeriatric service was fully implemented, whilst the duration (5 years) and number of patients for each group were similar. Varying the two groups by a year either side of this cut-off showed differences remained similar. It is possible that the COW model introduced in 2016 to the orthogeriatric service had additional impact of reducing LOS. In support for the choice of these two groups defined by the two periods of 2009–2014 and 2015–2019, our previous study using trend analysis of data for this cohort of patients showed that the proportions of prolonged LOS in hospital only started to decline significantly after the first period [15]. We recognise that there may be a lag period in outcome improvement after implementation of a certain component of the orthogeriatric service. Since the completion of this study, our hospital has brought out further initiatives based on an Integrated Discharge Bureau (IDB), a multidisciplinary team of nurses, social care professionals and discharge trackers who identify patients and inform them about accessing services that support their hospital discharge. The IDB, with the help of a newsletter, can inform patients and discharge teams about, for example: access to step-down beds in the community; a hotline to contact the complex discharge team; daily multidisciplinary team meetings to ensure timely discharge for patients who do not meet criteria to stay in hospital; training of ward staff to assess for care needs; special labels to prioritise blood tests for patients being discharged.

### Strengths and limitations

The strengths in this study lie in the wide range of important quality-care measures that were collected according to the NHFD audit programme protocol [8]. Data for all patients admitted with a hip fracture were documented in detail from the time of admission to discharge. The completeness and quality of data in this study were managed by an appointed senior orthogeriatrician and patients’ characteristics are similar to those reported in the NHFD audit [8]. The NHFD routinely records age and sex distribution, but not other sociodemographic factors or comorbidities. However, variables including pre-fracture mobility and types of surgical procedures are available which reflect underlying frailty and severity or complexity of hip fractures [27]. This allowed us to include these aspects as potential confounding factors in multivariable regression analysis of differences in LOS between years of study. There were certain limitations to the study including the relatively small number of patients discharged to residential and nursing care, but standardised data of bed days provide a useful estimate for national estimates. We did not collect information on discharge delay amongst patients who were deemed “medically-fit-for-discharge”. Several news sources have reported data, obtained through the Freedom of Information Act, showing a significant number of medically fit patients died while waiting for discharge [26]. Further studies are needed to assess outcome consequences of such patients since little information is available in the current literature. The present study also examined possible differences in hip surgeries and health expenditures over the years. We wish to point out that in our previous study of the same cohort of patients [15], we examined temporal trends for various care-quality indicators including prolonged LOS for the entire sample, *i.e.* irrespective of discharge destinations, an area of research that has not been well-documented. By contrast, the present study focussed on the impact of an orthogeriatric service on the LOS in patients waiting to be discharged to specific destinations including back to own homes, rehabilitation, residential and nursing care, in order to estimate the reduction in expenditure induced by service improvement.

In conclusion, complete implementation of an orthogeriatric service is associated with a reduction in LOS for all patients and cost-savings, but most notably those who were newly discharged to nursing or residential care.

### Supplementary Information

Below is the link to the electronic supplementary material.

Supplementary file1 (DOCX 321 KB)

## Data Availability

No additional data are available.
