# Supplementary material for: An orthogeriatric service can reduce prolonged hospital length of stay in hospital for older adults admitted with hip fractures: a monocentric study
Source: Aging Clin Exp Res. 2023 Nov 14;35(12):3137–46. doi: 10.1007/s40520-023-02616-3 (PMC10721690; doi:10.1007/s40520-023-02616-3)
Supplement: Supplementary file 1 — Supplementary file1 (DOCX 321 KB) [file 40520_2023_2616_MOESM1_ESM.docx]

**Supplementary Figure 1.** Distribution of length of stay in hospital showing right skewness.

**
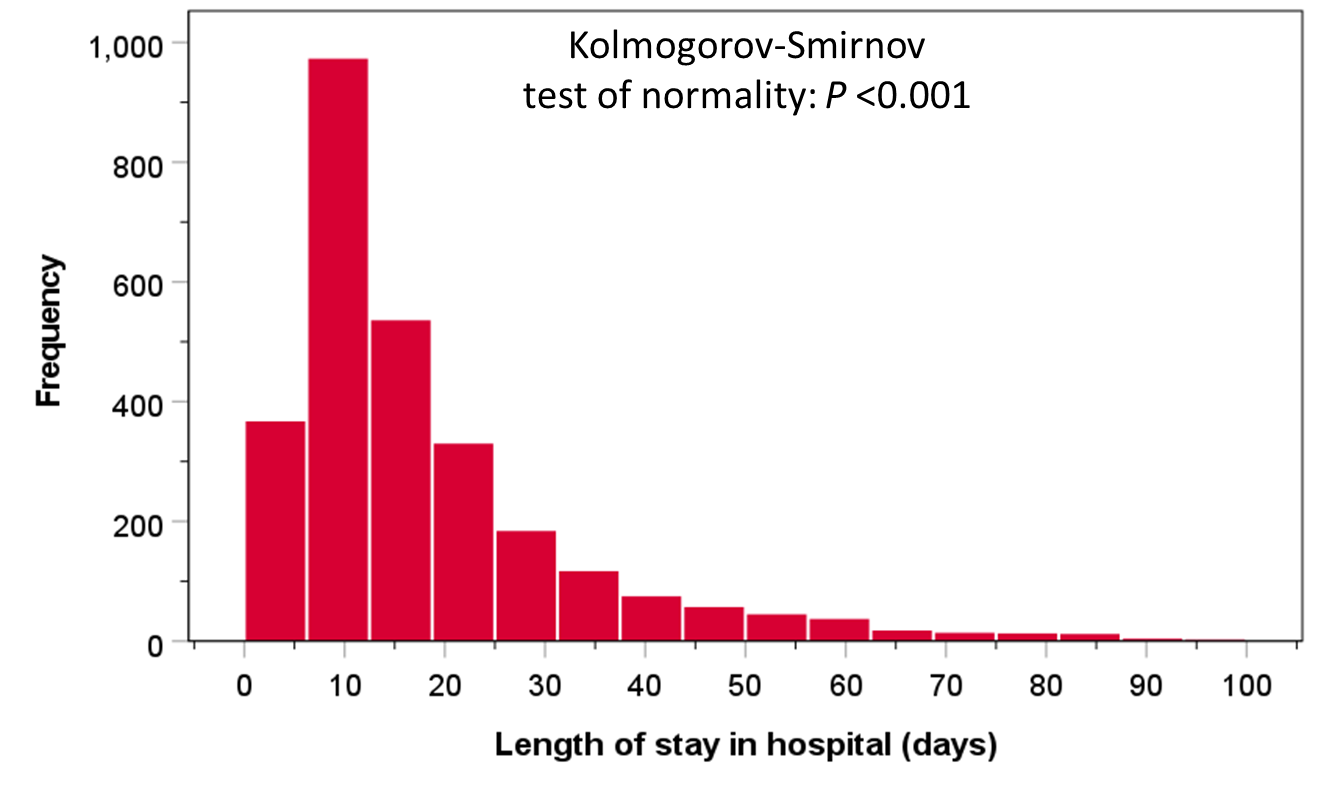
**

**Supplementary Figure 2.** Length of stay in hospital according to discharge destination.

**
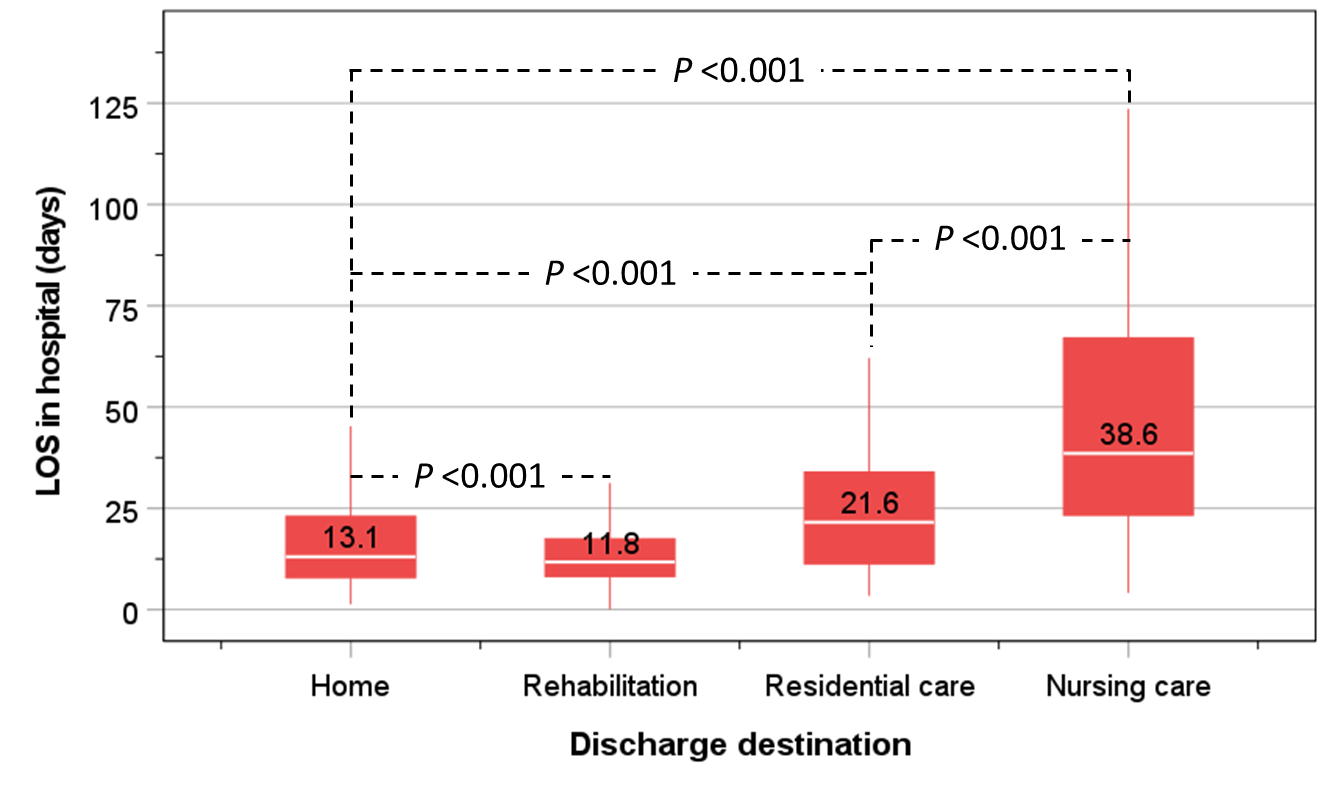
**

**Supplementary Figure 3.** Distribution of LOS in hospital before and after implementation of orthogeriatric care service. Kolmogorov-Smirnov test for differences in LOS between two periods: test statistic = 7.532, *P* <0.001. The curves indicate cumulative percentage sums for the respective study groups.

**
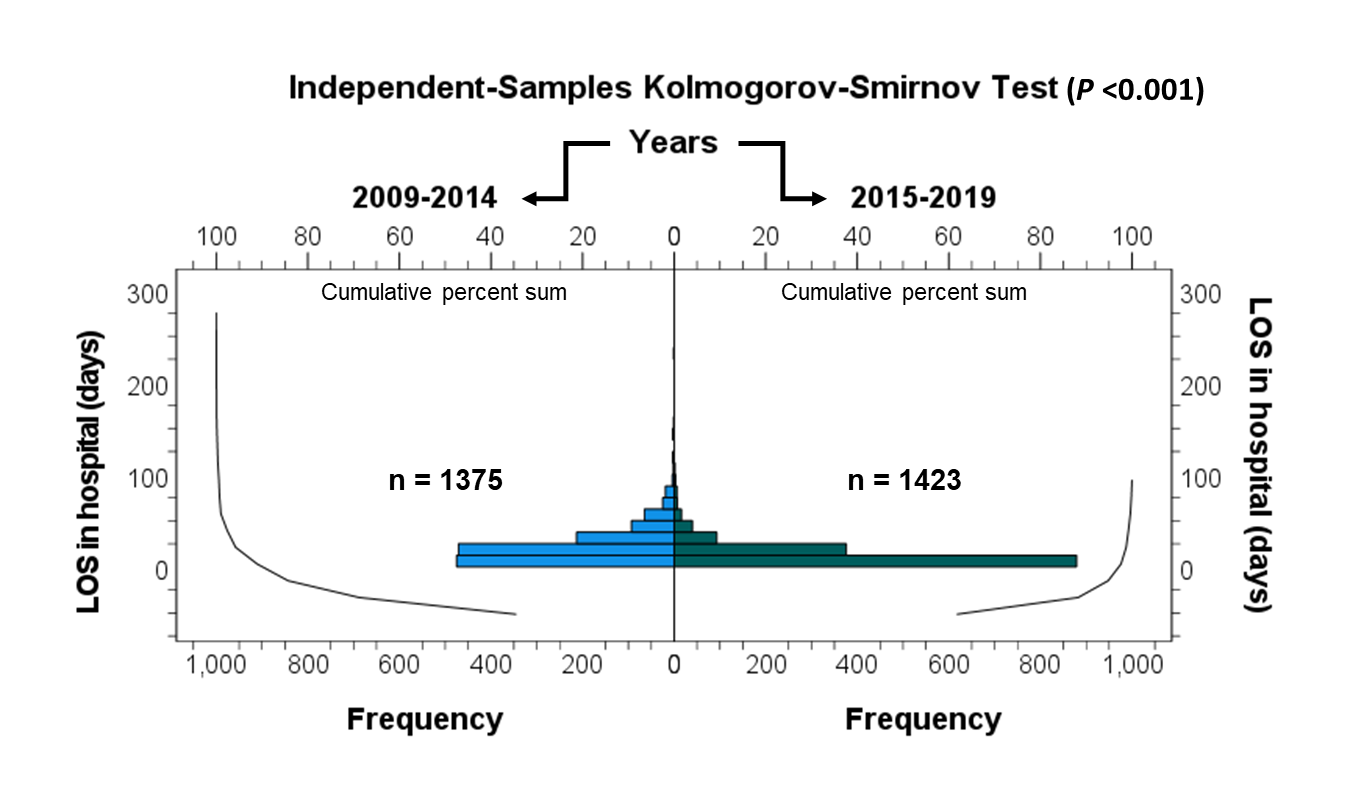
**
